# Twelve tips for starting and organizing a local Brain Bee anywhere

**DOI:** 10.12688/mep.20152.1

**Published:** 2024-02-15

**Authors:** Ernesto Navarro Garcia, Sebastian Leon, Gabrielle Walcott-Bedeau

**Affiliations:** 1 Physiology and Neuroscience, St George's University, Saint George's, Saint George, Grenada; 2 University of Central Florida, Orlando, Florida, USA; 3 NanoScience Technology Center, University of Central Florida, Orlando, Florida, USA

**Keywords:** Brain Bee, STEM, education, neuroscience, high school, graduate, volunteering, public service

## Abstract

The Brain Bee serves as an international platform providing high school students with opportunities for undergraduate and graduate-level education in neuroscience. This annual competition welcomes participation from high school students and schools, fostering both individual- and team-based competition in a dynamic and engaging manner. The assessment involves a primary multiple-choice and short-answer exam, a secondary neuroanatomical lab practical exam, and a final oral exam administered by affiliated faculty or coordinators. During the final oral exam, the top ten students are given three chances and simultaneously respond to the same question. They write their answers on individual whiteboards until only one student remains. This unique format not only adds an element of excitement, but also allows students to build confidence within themselves and connections within STEM fields. The importance of local Brain Bees is emphasized as winners from these chapters earn the opportunity to compete nationally, and potentially internationally. Throughout the academic year, local Brain Bees, led by college students and faculty, educate high school students about various neuroscientific fields and their STEM applications through interactive and engaging sessions. These sessions culminate in an annual competition where students explore the intricacies of the human brain, spanning human physiology, pathology, and behavior. The program also exposes students to anatomical models, research, and guest speakers within the field of neuroscience. To ensure the success and continuity of local chapters, our 12-tips article provides valuable advice for running a safe and smoothly operating Brain Bee program, promoting sustained participation and enthusiasm.

## Introduction

The Brain Bee program was founded by Dr. Norbert Myslinski of the University of Maryland, North America. This program has since evolved from a local competition to an international event that fosters neuroscience at an early age. The Brain Bee is a competition for top high school students across the state, nation, and ultimately, the world. They compete to win prizes and scholarships. For students to reach the international level, they must win their local and national Brain Bee. During the local event, students are exposed to neuroscience in all fields, from psychology to neurosurgery, and everything in between. This event provides access to high levels of education for all socioeconomic statuses and gender ranges ^1^. Although many high school programs offer extra-curricular activities, funding across schools and regions varies. This financial disparity can lead to unequal outcomes in student exposure to diverse fields. In addition to funding disparities, many extracurricular activities focus on sports and community services. While these aspects are an important component of high school curricula, early exposure to the graduate and medical levels of science is crucial for promoting future STEM involvement and scientific literacy. Furthermore, the Brain Bee event allows students to network with college faculty and others alike. The Brain Bee has many benefits for all those involved. Young students who participate develop a deeper sense of how science is conducted, increase their interest in STEM-related careers, and allow them to build long-term relationships with mentors in the field ^2, 3^. While the Brain Bee is a relatively new event, its continuous growth across the globe allows participants to develop in social and academic aspects. Likewise, university volunteers benefit from this event by being given the opportunity to give back to the community, practice their didactic teaching skills and mentors, and build a larger network within the field. As previous coordinators of successful Brain Bees in Orlando and Jacksonville, Florida, and St. George, Grenada, we offer our 12 tips to organizers of local and future chapters by providing information on how to make a fun, interactive, and beneficial event.

### Tip 1: familiarize yourself with the Brain Bee

If you are well acquainted with Brain Bee, first, we want to say, Thank you! You are part of an expanding and fascinating field. Your contributions may help build future scientists. Please head to ‘Tip 2.’

If you are a newcomer, welcome! Before planning an event, you must be familiar with it. Please see www.thebrainbee.org/competition/. Here, you will find all the information you need to know about the Brain Bee. If you are ready, let’s get to planning.

### Tip 2: gather a strong volunteering force to help you manage the event

Creating and managing a Brain Bee is not a one-person job. Get in touch with a local university and reach out to a Registered Student Organization (RSO) in the Biomedical or Psychology department, preferably in the field of neuroscience. While this is not a must, it will be easier to set the event in motion. Set up a meeting to explain to the RSO/university students what the Brain Bee is about and how they can get involved. Have a presentation ready to tell them how they will benefit, how they can help, and what is expected of them. Having an undergraduate volunteer force is crucial for managing workloads and efficiently delegating roles. Additionally, a large number of volunteers will be necessary to teach and conduct the local main event. If a sufficiently large body of volunteers is not available, alternatives such as group Zoom meetings or similar are an effective option. Students, along with their parents and their teacher chaperones, can join a centralized Zoom meeting at scheduled times for a group lecture or discussion. This method is especially effective if schools are too far away to travel to, if individual students are not participating associated with a local high school, or if there is an insufficient volunteer-to-student/school ratio. We encourage you to be creative in making this event inclusive and accessible to everyone who wishes to participate.

### Tip 3: find a sponsor in the university

After you partner with an RSO, find one or more professors within the university who are willing to be sponsors and assume a leadership role. Having professors within the university play an active role in ensure a direct affiliation with the university and add legitimacy to your local chapter. Furthermore, their participation will draw more interest from the student body, faculty, and staff. Additionally, their expertise in the topic and academia can benefit you as they can talk about their research, invite guest speakers, and add more components to your main event.

### Tip 4: gather schools to participate

This step is arguably the most challenging. Reaching out to high schools can be done in several ways. Be patient and persistent. Schools will inherently benefit from the competition, as it brings free higher education and provides opportunities for their students they otherwise would not have. We find that most schools are willing to participate when they are shown how their community and students will benefit. We also found that schools are more likely to participate if successful events have occurred in the past. The following are some ways to achieve this:

**Email or call** the high school superintendent (the director of all high schools; this is very common in the United States of America as schools are administrated by counties within a state). They can help you by sending your ideas in a mass email to all the high schools they oversee. Always check your region’s requisites and policies and remember you are engaging with underage students. 

**Email** all school principals a PDF/PPT about the event and set a meeting time. This is time-consuming, and you may get many rejections, but the more schools you reach out to, the more students you will have at your event. In turn, the more schools successfully participating will result in more schools willing to engage in the next year’s event. When you email, always CC your university sponsor and add your credentials when you sign your email. This will add validity to your email and increase the likelihood of a response. If a school chooses not to participate, reach out after the first event, provide documentation of other participating schools, and ask them to reconsider participating in the next academic school year.

**Call** as many schools as you can and ask to speak with the principal. This is very efficient if the principal takes your call, and you can briefly explain your event and set up a meeting to elaborate.

These methods of reaching out to high schools have worked, however, feel free to be creative in how you reach out to them. Remember, the overall goal is to gather as many schools as possible; in this way, the impact of your Brain Bee increases.

### Tip 5: register your Brain Bee chapter

To make your chapter legitimate and yearly national and international competitions, you must register your Brain Bee. Head to www.thebrainbee.org/about/ and find the current director’s contact information. In the email, add your country, state, affiliated university, sponsor, and the current state of your Brain Bee. The current state should contain information regarding the number of high schools, participants, volunteers, and related events that are linked to your chapter. See ‘Tip 11’ for linked events.

### Tip 6: gather your teaching materials

A large portion of the success of the Brain Bee is linked to the teaching aspect that occurs prior to the international competitions. Your volunteer force will need to be efficient and well prepared to teach avid neuroscience enthusiasts, many of whom have a wide knowledge of neuroscience. For starters, head to www.brainfacts.org/the-brain-facts-book. The Brain Facts book is free and is used as the main curriculum for most, if not all, Brain Bee chapters across the globe. You can also use https://brain.mcmaster.ca/BrainBee/Neuroscience.Science.of.the.Brain.pdf. This book was provided by the Brain Bee committee and supplements the Brain Facts Book. Both resources are free. Some content variations may exist, and they are truly dependent on what you, the organizer, want to add. Make the powerpoints of each chapter and have your volunteers read the chapters and be ready to teach. Preparing your materials is time consuming. Do this well ahead of your starting date and reach established chapters for help if needed. Utilize any free resources that increase the student’s interest and learning preferences and be mindful of maintaining equal access to all participants. This is particularly important in underserved places, where not every student may have access to technology. In Orlando and St. George’s chapter, the use of books, visual aids, charts, images, and videos has proven successful. Other useful resources that you may want to use are:

The University of British Columbia has a fantastic neuroanatomical website that is perfect for all levels ( www.neuroanatomy.ca).

The Neuroscience Alliance at the University of Central Florida has a large YouTube channel with video lessons based on the Brain Facts Book ( www.youtube.com/brainbee)

The Apple Store will have a free neuroscience application that will be released by the middle of 2024. Stay tuned for **Neuroscience by Hive Mind Ed**.

### Tip 7: set out a timeline to begin teaching and finalize your curriculum

Your Brain Bee is likely to operate based on the date of the national and international Brain Bee. This date will be communicated via email. Plan dates to teach and set a date for the main local event. Teaching can be done in many ways and varies from one chapter to another. This is likely to occur as an extra-curricular activity; your volunteers will be teaching after high school hours either in person or online. This can be achieved in several ways, and we list what we have tried with success.

One method is to send volunteers to teach in person at the participating high schools. This is our preferred method, as active learning has been proven to be most beneficial ^4^. Students will be more engaged, actively participate, and form relationships with you or your volunteers. This usually affects your turnout positively as more high school students participate.

The post-pandemic landscape has introduced a mixed bag of regulations that have both adversely impacted and brought about benefits for everyone. One notable positive aspect is the newfound ability to conduct numerous online events. While this may not be the ideal scenario, the online format proves to be an invaluable resource. Hosting sessions through platforms such as Zoom, Skype, or any other free video conferencing tool offers flexibility and convenience. However, it is crucial to ensure equal access to the underserved population.

### Tip 8: plan your main event

The main event is when you see all your efforts to come to fruition. Here, high school students will come and compete for a place in the national brain bee. Think of how you will go about testing the participants. This can be done with multiple choice tests, round robins, a practical lab, etc. Be creative! These events must be prepared in advance. Plan activities, food, speakers, prizes, and other logistical needs before you plan to host an event. If you plan to host an event at your local university, request a space where you can fit all your expected turnout. Most universities have been booked for several events. Reach out early and reserve a building, salon, or any site where the event can take place and flow easily. If you are planning to have a keynote speaker, ask for various candidates in advance. Calculate the cost of food and beverages for guests. Often, companies give ‘student discounts’ or ‘large order’ discounts. Make a list of several vendors you have in mind: call and ask about prices and other options. Maximize your budget. Always be mindful of people’s diets. If you go for generic food (e.g., pizza), let guests know in advance, some may have to bring their own food.

You may also want to reward participants and winners. If possible, plan to purchase medals, trophies, and print certificates. This reward system will motivate your students to participate and will also help them prove their participation. All certificates for participating students were hand-signed by our lead coordinator and the head of Neuroscience at the University of Central Florida, who served as our university sponsor. An original idea from the Orlando chapter is the ‘travelling trophy. ’ This trophy is given to the high school that contributed the most students to the top 10, and the trophy gets passed around every year to the winning institution and a small plaque is engraved. Over the years, you will see many plaques in your trophy. For funding ideas, head to ‘Tip 10.’

### Tip 9: building your tests to have a local winner

This is a competition, and there must be a winner. Ultimately, this is how students have the opportunity to participate in the National Brain Bee and then the International Brain Bee. As organizers, you must have a system to decide who wins. Use the free resources of ‘Tip 6’ to make a multiple-choice exam. This is the simplest and most efficient method for testing the winner’s knowledge. However, a single test may be underwhelming. You may have more than one! Here is an example of how the Orlando chapter operates:

**Phase 1**: Students take one 50 multiple choice exam, with questions from the resources you have used from tip 6. Print out extra copies; you want to be prepared.

**Figure 1.**
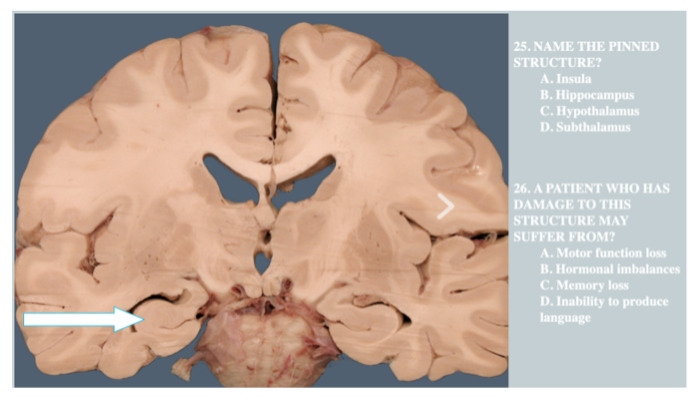
sample item from lab practical at Orlando Brain Bee.

**Phase 2**: Students take one 35 question lab practical based on anatomy and the function of such anatomy. Figure 1 shows a sample question utilized in the lab practical at the Orlando competition.

To obtain the top 10, pool scores from Phases 1 and 2 from each student, generate an average, and select the top 10. Here, your volunteer force will help administer the tests and grades. The top 10 students then proceed to Phase 3.

**Phase 3**: The top 10 students are asked questions in front of the audience by a panel of neuroscientists. The students proceed to write their answers on a personal white board and show their answers at the end of 1 minute timer. The students are then filtered out when they answer questions incorrectly until there is one winner.

### Tip 10: generate funding and bring attention to your event

Local Brain Bees are relatively inexpensive; however, they require some money. This funding will help you pay the costs of the food and prizes, and perhaps allow you to give a small scholarship to the winner. Your affiliated university may give you some money, contact SGA, and ask for RSO funding. Reach out to doctor offices, large companies, etc. Some may be willing to donate; in return, they may ask you to advertise their practice during the event. Organize a food drive, sell merchandise, do carwashes, and be creative! There are plenty of ways to generate money. Always be ethical and mindful. Furthermore, do not advertise prizes or scholarships until you are sure that you will have the funds to comply. Advertising your event is also important, and this will bring attention to your event in other neighboring schools and regions. In turn, you may receive interest from other schools willing to participate. Try reaching out to the local news, the university’s journalism department, and consider opening a social media account.

### Tip 11: make your Brain Bee main event unique with parallel events

To generate more interest and turnout in your main event, plan parallel activities along the Brain Bee. To maximize attendees’ benefits, you may want to add other workshops along the way. These workshops work best during testing phases 1 and 2 and lunch time. While high school students take their examinations, friends and family can attend these workshops to learn more about neuroscience. For example, the Orlando chapter had an EEG workshop, a basic neuroscience course for newcomers and adults, and a mini-research symposium. High school, undergraduate, graduate, and medical students were invited to present their research via poster presentations during lunchtime. This mini-symposium helped foster a research spirit among the high school participants. This symposium gives them an idea of the current research and how to prepare for research days. Furthermore, this helps presenters share their research and gives them an environment in which to practice their presentation skills.

### Tip 12: choose a successor

The Brain Bee will continue to exist beyond the event that you are organizing. Choosing a suitable successor that is passionate about this will ensure your chapter lives long after you move on. Talk to your volunteers and get to know them. Eventually, a few of them will show you that they are prepared to carry on this legacy.

## Conclusion

The impact of the Brain Bee is well documented, and its impact can be seen throughout the many events that occur at the international level. The continuous growth of the Brain Bee program is largely due to a volunteer population that has a passion to teach and expand the field of neuroscience. These 12 tips will ensure that anyone who shares an interest in the Brain Bee can manage to start a local chapter and build it towards the international platform. The choice of tips given here are from experienced individuals who have been successful in starting and maintaining various chapters across different regions. This is particularly important, as it states the different challenges of working in both developing and underdeveloped countries, where funding and resources vary. The overall idea of how to build a local chapter is thoroughly described, and various ideas and steps have been discussed based on prior experiences. Altogether, this is a great steppingstone for anybody interested in becoming a part of the Brain Bee.

## Data Availability

No data are associated with this article.
