# Supplementary material for: Twelve tips for starting and organizing a local Brain Bee anywhere
Source: MedEdPublish (2016). 2024 Feb 15;14:5. [Version 1] doi: 10.12688/mep.20152.1 (PMC11333873; doi:10.12688/mep.20152.1)
Supplement: Peer review history

# Reviewer report: Reviewer response for version 1

**DOI:** 10.21956/mep.21577.r36160

**Published:** 2024-08-19

**Authors:** Amelia Adcock

**Affiliations:** 1 Department of Neurology, West Virginia University, Morgantown, West Virginia, USA

Very well written, complete and motivating game plan to help any interested party participate in the Brain Bee.

 Good introduction to the history and spirit of the Brain bee including it's role in positively addressing social determinants of health-type disparities in preparedness for graduate or undergraduate school application among HS students. 

If evidence from practice is presented, are all the underlying source data available to ensure full reproducibility?

Yes

Is the topic of the practical tips discussed accurately in the context of the current literature

Yes

Are all factual statements correct and adequately supported by citations?

Yes

Are the conclusions drawn balanced and justified on the basis of the presented arguments?

Yes

Are arguments sufficiently supported by evidence from the published literature and/or the authors’ practice?

Yes

Reviewer Expertise:

Medical education, geographic healthcare disparities, rural stroke, telestroke

I confirm that I have read this submission and believe that I have an appropriate level of expertise to confirm that it is of an acceptable scientific standard.

# Reviewer report: Reviewer response for version 1

**DOI:** 10.21956/mep.21577.r37084

**Published:** 2024-06-14

**Authors:** Leandra Fernandes Procópio

**Affiliations:** 1 Autonomous University of Madrid, Madrid, Spain

The article "Twelve Tips for Starting and Organizing a Local Brain Bree Anywhere" offers a detailed guide on how to establish and manage a local chapter of Brain Bree based on previous successful experiences.

 The article presents a formal, accessible, and well-structured style, allowing the reader to follow the content without any difficulty. However, the authors should make some minor modifications to achieve an excellent production. Result: The introduction presents statements that are not supported by bibliographic citations to confirm them. The lack of solid rigor makes it difficult for the reader to trust the claims being made.

- *The Brain Bee program was founded by Dr. Norbert Myslinski of the University of Maryland, North America. This program has since evolved from a local competition to an international event that fosters neuroscience at an early age*
- *This financial disparity can lead to unequal outcomes in student exposure to diverse fields. In addition to funding disparities, many extracurricular activities focus on sports and community services. *

It presents no empirical evidence; the article lacks data on the positive impact on students. The authors should provide participation statistics or offer data on the demographic diversity of the participants. Additionally, the following claims need to be supported by evidence:

- *While the Brain Bee is a relatively new event, its continuous growth across the globe allows participants to develop in social and academic aspects*
- *The impact of the Brain Bee is well documented, and its impact can be seen throughout the many events that occur at the international level*

The authors of the article could include practical examples of how Brain Bee has impacted the lives of participants who have pursued careers in the field of neuroscience.

 This article has a clear focus on inclusion; however, it does not mention specific solutions or strategies for schools that are less advantaged in terms of access to technology, funding, or resources, as it is possible that not all can successfully implement a local chapter of Brain Bee.

 As a suggestion, a final tip on collecting feedback from participants to evaluate their experience could be introduced. This feedback can improve the outcomes of future events and create a more effective and successful impact.

 It is worth noting the abundance of links to neuroscience resources for preparing for Brain Bee. However, to make these resources accessible and uphold the principle of inclusion, they should be available in multiple languages for students whose first language is not English.

 When reading the text, it would be helpful to specify the number of volunteers needed to manage the event.

 In summary, the article requires minor changes, as specified above, to be fully approved.

If evidence from practice is presented, are all the underlying source data available to ensure full reproducibility?

Partly

Is the topic of the practical tips discussed accurately in the context of the current literature

Partly

Are all factual statements correct and adequately supported by citations?

No

Are the conclusions drawn balanced and justified on the basis of the presented arguments?

Partly

Are arguments sufficiently supported by evidence from the published literature and/or the authors’ practice?

No

Reviewer Expertise:

Neurociencia en Educación, Formación del profesorado, práctica pedagógica, educación inclusiva, identidad docente, Altas Capacidades, estrés y burnout docente.

I confirm that I have read this submission and believe that I have an appropriate level of expertise to confirm that it is of an acceptable scientific standard, however I have significant reservations, as outlined above.

## References

[1] : Twelve tips for starting and organizing a local Brain Bee anywhere. *MedEdPublish* .2024;14: 10.12688/mep.20152.1 10.12688/mep.20152.1 PMC1133387339165589

# Reviewer report: Reviewer response for version 1

**DOI:** 10.21956/mep.21577.r36387

**Published:** 2024-04-16

**Authors:** Alba Peris-Yague

**Affiliations:** 1 Universidad Politecnica de Madrid, Madrid, Community of Madrid, Spain

The article *Twelve tips for starting and organizing a local Brain Bee anywhere* by Navarro Garcia et al, provides a set of tips on how to plan and put in place a local Brain Bee competition based on the authors’ previous experience.

 This article is well-written and has potential to provide insightful tools on how to organize a Brain Bee competition, however, there are a few aspects that could be revised.

 The authors are experienced in organizing multiple Brain Bee events and this article could benefit of supplementary material that provided tools for educators to implement in their new local chapters. If possible, the authors could share such material.

**Introduction:**

 While the introduction brings up interesting and important points that are relevant for the article, the authors could consider providing citations to some of the claims made as well as provide some examples in some statements.

  

1. The authors could introduce the Brain Bee platform (i.e. the link to the site) at the beginning and provide a citation to the following information provided: *The Brain Bee program was founded by Dr. Norbert Myslinski of the University of Maryland, North America.  This program has since evolved from a local competition to an international event that fosters neuroscience at an early age.*

Furthermore, the authors could be more specific with the statement ‘’at an early age’’ and provide an age range.

  

1. The statement: *The Brain Bee is a competition for top high school students across the state, nation, and ultimately, the world. *The authors could re-consider this statement as the mission of the Brain Bee is to motivate and inspire students to learn about and pursue careers in neuroscience ( https://www.thebrainbee.org/about/), not to provide a competition for top high school students.

 

1. At the moment it is unclear how the Brain Bee relates to the following statement: *This event provides access to high levels of education for all socioeconomic statuses and gender ranges.* The citation provided with this statement does not discuss how the Brain Bee specifically provides what the statement suggests.

 

1. The authors could provide a citation and expand when discussing that financial disparity can lead to unequal outcomes in student exposure to diverse fields. In their experience and/or based on other studies, it would be great if the authors discussed in more detail how could the Brain Bee specifically help provide an equal opportunity setting for learning about Neuroscience.

 

1. The following statement is lacking a citation: *While these aspects are an important component of high school curricula, early exposure to the graduate and medical levels of science is crucial for promoting future STEM involvement and scientific literacy. *Furthermore, the authors could be more specific when referring to ‘’early exposure’’.

 

1. The authors could provide a citation to the following statement and provide some data to exemplify the growth of the Brain Bee: *While the Brain Bee is a relatively new event, its continuous growth across the globe allows participants to develop in social and academic aspects.*

**Tip 2: **

1. The authors suggest having a presentation to gather volunteers. If possible, the authors could provide a sample presentation used based on their experience.
2. The authors could provide an estimate number of volunteers needed based on their experience and specify how many volunteers are typically needed per student.

**Tip 4**:

1. The authors could also provide as a supplementary material a suggestion of an e-mail or telephone pitch when contacting high schools that have found to be successful in the past.
2. If possible, when the authors suggest *‘Email all school principals a PDF/PPT about the event’*, they could consider sharing an example file that they have used in their events.

**Tip 6: **

1. The authors provide some resources that are very valuable for the goal of this article. However, the authors do not acknowledge that most resources are in English. Here and / or throughout the manuscript, the authors could provide some insight on how to reach students beyond the English-speaking world.

**Tip 7:**

1. If available, the authors could provide as supplementary material a suggested timeline / syllabus that provides an example of a successful timeline for preparation.

**Tip 9: **

1. In line with the comment above, the authors could provide a sample timeline for the tests. How much time do they suggest to happen between phase 1 and phase 2 tests? Do they occur on the same day at different timepoints? If possible, the authors could provide a sample day-of-testing schedule with their suggestions of when best to include other activities, breaks, etc.

**Figure 1.**

1. The figure title is *sample item from lab practical at Orlando Brain Bee*. However, the authors have not discussed what ‘lab practical’ are with relation to Brain Bee. Perhaps the authors could dedicate a section in their introduction to provide a specific introduction on the types of Brain Bee teaching materials and overall organization.

**Tip 10:**

1. The authors could provide some local, national and / or international funding suggestions / grants.
2. If possible, the article would benefit from a sample supplementary material of a breakdown of needed funding in the authors’ experience.

**Conclusion**

1.  The statement: *The impact of the Brain Bee is well documented* appears to be somewhat vague. The authors should provide citations that the readers could refer to.
2. If available, the authors could provide a list and metrics of success of their previously organized Brain Bee competitions that the readers could refer to.

If evidence from practice is presented, are all the underlying source data available to ensure full reproducibility?

Partly

Is the topic of the practical tips discussed accurately in the context of the current literature

Partly

Are all factual statements correct and adequately supported by citations?

No

Are the conclusions drawn balanced and justified on the basis of the presented arguments?

Partly

Are arguments sufficiently supported by evidence from the published literature and/or the authors’ practice?

No

Reviewer Expertise:

Memory, iEEG, neuroscience outreach and education

I confirm that I have read this submission and believe that I have an appropriate level of expertise to confirm that it is of an acceptable scientific standard, however I have significant reservations, as outlined above.
